# The Effect of Sleep Curtailment on Hedonic Responses to Liquid and Solid Food

**DOI:** 10.3390/foods8100465

**Published:** 2019-10-10

**Authors:** Edward J. Szczygiel, Sungeun Cho, Robin M. Tucker

**Affiliations:** Department of Food Science and Human Nutrition, Michigan State University, East Lansing, MI 48824, USA; szczygi7@msu.edu (E.J.S.); chosunge@msu.edu (S.C.)

**Keywords:** sleep curtailment, hedonics, complex food matrices, sweet liking phenotype, sweet taste, texture

## Abstract

It is currently unclear whether changes in sweet taste perception of model systems after sleep curtailment extend to complex food matrices. Therefore, the primary objective of this study was to use a novel solid oat-based food (crisps) and oat-based beverage stimulus sweetened with sucralose to assess changes in taste perception after sleep curtailment. Forty-one participants recorded a habitual and curtailed night of sleep using a single-channel electroencephalograph. The next morning, overall sweetness, flavor, and texture liking responses to energy- and nutrient-matched oat products across five concentrations of sweetness were measured. Overall (*p* = 0.047) and flavor (*p* = 0.017) liking slopes across measured concentrations were steeper after curtailment, suggesting that sweeter versions of the oat products were liked more after sleep curtailment. Additionally, a hierarchical cluster analysis was used to classify sweet likers and non-likers. While the effect of sleep curtailment on sweet liking did not differ between sweet liking classification categories, sleep curtailment resulted in decreased texture liking in the solid oat crisps for sweet non-likers (*p* < 0.001), but not in the oat beverage. These findings illustrate the varied effects of sleep on hedonic response in complex food matrices and possible mechanisms by which insufficient sleep can lead to sensory-moderated increases in energy intake.

## 1. Introduction

There is a growing body of evidence that insufficient sleep can alter taste perception. Several recent psychophysical studies have reported that short sleep duration is associated with increased preferred sucrose concentration [1,2,3] and increased perceived intensity of sour and umami taste [4]. Insufficient sleep-induced changes in taste perception may partially moderate the well-supported relationship between short sleep duration, increased dietary intake of highly palatable high-carbohydrate and high-fat foods, and weight gain [5,6,7]. Brain imaging research suggests that insufficient sleep results in increased neural sensitivity to the reward properties of food [8,9,10,11,12]. This heightened sensitivity may increase the consumption of palatable food for pleasure, also known as hedonic eating. Hedonic eating is thought to promote weight gain, as highly palatable food tends to be energy-dense [13]. Sweetness is commonly associated with the palatability of food [14] and when tasted, initiates brain reward processes [15]; therefore, sweet taste is of particular interest when exploring relationships between insufficient sleep and hedonic eating. Nearly 40% of the US adult population is reported to sleep less than the recommended 7 h per night [16] and nearly 40% of American adults suffer from obesity [17]. A similar prevalence of insufficient sleep has been documented globally [18,19]. Therefore, understanding the mechanisms by which insufficient sleep can lead to weight gain is of importance to scientists. Furthermore, these mechanisms may provide deeper insight regarding food choices and food acceptance and therefore, are of particular interest to both the food industry and public health advocates.

Very few studies utilize complex food when examining the effect of insufficient sleep on taste function [4,20]. Instead, nearly all existing sleep-taste research has been conducted using model systems—prototypical tastants dissolved in deionized water—and evaluated while wearing nose clips [1,2,3,4,21,22]. Results from previous psychophysical studies examining the effects of sleep on taste perception need to be replicated in more complex food matrices as findings in model systems do not always align with findings using complex foods [23,24,25,26]. The simplicity of model systems allows participants to evaluate taste with minimal distraction from other sensory inputs like texture or aroma, but affective judgments of foods and beverages are determined using all senses, including appearance, mouthfeel, auditory characteristics, geometry, and the physical state of food [27]. Thus, further efforts are needed to assess the generalizability of taste-related findings from psychophysical studies to complex food matrices. 

In addition to the general issues discussed above regarding translating findings from model systems to food, there are particular reasons to believe that the generalizability of findings from model stimuli to complex foods under conditions of insufficient sleep could be especially problematic. In the context of complex food, research suggests two important effects of insufficient sleep that could alter perception: impaired sensory neural processing [28,29] and increased somatosensory sensitivity [30]. First, given that the orbitofrontal cortex (OFC), often described as the neural control center for appetite [31], is impaired after sleep curtailment, the ability to interpret multimodal information may be compromised [28,29]. Under normal conditions, processing of specific attributes within multimodal sensory information is already limited. For example, when consuming complex foods, the ability of participants to separate perceived sweet taste liking from perceived flavor or overall liking may be diminished due to sensory interactions [32]. Thus, after sleep curtailment, impairment of OFC activity may result in further differences between perception in controlled systems and complex food systems [32]. The second concern about the generalizability of findings from model systems to more complex food matrices under insufficient sleep conditions stems from documented changes in somatosensory perception. Sleep curtailment has been implicated in acute reward system-mediated hyperalgesia—an increased sensitivity to pain [33]—and increased oro-facial somatosensory sensitivity, particularly the tongue [30]. While speculative, increased hyperalgesia and increased oro-facial sensitivity might decrease the acceptability of the texture of crispy or crunchy solid foods and increase preference for softer foods, semisolids, or beverages that require less oral processing. In summary, processing of sensory information, reward processing of that information, and changes in oral sensory sensitivity all represent opportunities for insufficient sleep to affect hedonic food perception. 

Individual differences in hedonic response to taste make it challenging to study the relationship between insufficient sleep and gustatory perception. Despite being an innately palatable taste at birth [34], liking responses to sweet taste as the concentration of sweetness increases differ across individuals. Three fundamental patterns of liking over a range of sweetness levels have been identified previously: sweet likers, who display a rise in liking as sweetener concentration increases; inverted U-shape responders, who show an increasing liking pattern up until a certain concentration before beginning to show a decrease; and dislikers, who display a reduction in liking as concentration increases [35,36,37]. Additionally, a fourth pattern where hedonic response to sweetness is the same regardless of sweetness concentration has been reported [36], but others have reported not observing these phenotypes [38,39]. These fundamental patterns of liking are partially determined by genetic factors [40,41], and thus, they are commonly described as “sweet liking phenotypes” (SLP). Sweet likers differ in expressed behaviors compared to the other phenotypes, including increased intake of sugar and sugar-sweetened beverages [42,43]. These behavioral traits suggest that sweet liking phenotypes are heritable indicators of general brain reward processing alteration. Given that the central hypothesis of this research is that insufficient sleep-induced reward processing alterations may influence hedonic perception of food, individual differences in response to sweet taste are an important factor to consider, as these baseline differences in reward processing may reduce the effects of insufficient sleep on the brain reward processing. While our previous work found that preferred sweetener concentration was similarly increased across sweet liking phenotypes after sleep curtailment [21], this relationship has not been evaluated in complex foods. Therefore, the question of whether SLP is an important factor moderating the effect of sleep curtailment merits further investigation in the context of complex foods. 

The main objective of this study was to evaluate changes in hedonic response to two complex sucralose-sweetened foods across a range of sweetness levels after a habitual and curtailed night of sleep and to compare these responses to a model system consisting of sucralose solutions. It was hypothesized that hedonic perception in the model system would change in accordance with our previous findings [21], that is, after a night of sleep curtailment, preferred sucralose solution concentration would be increased relative to hedonic response after a night of habitual sleep and a non-significant increase in the steepness of the slope of liking over a range of concentration would be observed. While it was expected that changes in patterns of sweetness liking would agree with our previous findings that showed that sleep curtailment increased the rate of liking as sweetener concentration increased, it was hypothesized that the change in pattern would be more pronounced when tasting “real” foods instead of sweetener solutions due to altered processing of multi-modal sensory information. It was also expected that broader hedonic measures, such as a flavor and overall liking, would show increases corresponding with increasing sweetness after sleep curtailment, as these terms have a greater potential to capture changes in multisensory perception unique to complex foods. Furthermore, it was hypothesized that sleep curtailment would result in decreased texture liking in a solid food and increased liking in a liquid food. A secondary objective was to assess if food form and SLP interact with sleep curtailment to alter sensory perception of complex foods. Although SLP was not found to differentially moderate changes in hedonic perception in model systems under conditions of insufficient sleep, we sought to confirm this finding in complex foods. It was expected that SLP would not moderate changes in hedonic perception of food after sleep curtailment, in accordance with previous work [21].

## 2. Materials and Methods

The protocol for this study was approved by the Michigan State University Human Research Protection Program (East Lansing, MI, USA). Written informed consent was obtained from all participants and a cash incentive was provided for study completion.

### 2.1. Participants

Participants between the ages of 18 and 45, without obesity (BMI < 30.0 kg/m^2^) or diagnosed sleep conditions, who typically slept 7–9 h per weeknight, and who had a consistent weekday bedtime were eligible to participate in the study. Participants were pre-screened using two criteria. First, each participant sampled both the oat “beverage” and oat “crisp” products (see Development of Stimuli section, below) evaluated in the study (sweetened with sucralose at the middle 0.032% *w*/*v* level) and were asked to rate their overall liking of each on a 9-point hedonic scale (extremely dislike (1) to extremely like (9)). The mid-range sweetness product was selected to avoid disproportionally recruiting sweet likers. Participants who rated either sample <6 (like slightly) were not eligible for the study to ensure that products could be considered generally palatable. Secondly, each participant sampled the highest concentration of sucralose in water (0.094% *w*/*v*) used in the study and was asked to report if they tasted any bitterness. Sucralose does not ordinarily display high levels of bitterness [44]. Nevertheless, participants who are extremely sensitive to bitterness [40] may find it challenging to evaluate sweetness in sucralose solutions. To avoid this, participants who tasted bitterness in the highly sweet sucralose sample were excluded from the study. Three individuals who were otherwise eligible were excluded due to tasting bitterness, and two were excluded due to dislike of the oat beverage. 

### 2.2. Development of Stimuli

Sucralose was selected as the sweetener for this study due to its sensory and functional properties. Sucralose has a taste profile with a similar character to sucrose and has low bitter and off-tastes compared to other high-intensity sweeteners [44]. Sucralose requires very small amounts to achieve the same sweetness as sucrose [45]. This property of sucralose enabled formulation of complex food products that varied in sweetness while minimizing changes in other sensory attributes, such as texture. The iso-sweet concentration and sweetness range were based on a similar study [21], which found these concentrations sufficient to observe changes in liking patterns after sleep curtailment. Briefly, a preliminary study was carried out to assess iso-sweet concentrations of sucralose compared to sucrose using the magnitude estimation methods of Reis et al. 2016 [46]. Sucralose concentrations of 0.004%, 0.011%, 0.032%, 0.060%, and 0.094% *w/v* were selected based on the magnitude estimation data power functions developed using data collected from fifty naive participants. These concentrations are equal in sweetness to 3%, 6%, 12%, 18%, and 24% *w*/*v* sucrose, respectively. The selected concentrations were found to be iso-sweet when used in a previous sleep-taste study using sweetener solutions [21]. To assess the effect of sleep curtailment on patterns of liking of complex food matrices, two energy and macronutrient-matched oat-based products were developed. Oats were selected as a versatile base ingredient because they can be used to produce similarly nutritious and palatable, solid, semi-solid, and liquid foods consistently across batches. The first product, an oat “beverage”, was developed to assess the effect of sleep curtailment on hedonic perceptions of liquid food, and the second product, an oat “crisp”, was developed to assess the effect of sleep curtailment on hedonic perceptions of solid food. The two products contained the same ingredients: whole grain rolled quick oats (Quaker Oats Company, Chicago, IL, USA), pure sucralose powder (Sweet Solutions, Edison, NJ, USA), and filtered water (Besco, Battle Creek, MI, USA). In both products, sucralose was added to water at the concentrations discussed previously and used to produce five differently sweet versions of both products. A proximate analysis was performed by Great Lakes Scientific (Stevensville, MI, USA) using the Association of Analytical Chemists (AOAC) method. A breakdown of the macronutrient content per 100 kcal is displayed in Table 1. The two products were matched on macronutrients per kcal. The only major difference between the two products was the moisture content, as designed. 

Oat beverages were produced by creating an oat slurry by blending (Nutribullet, NutriLiving, Northridge, CA, USA) 240 g of sucralose-sweetened water and 50 g of whole grain rolled quick oats (Quaker Oat Company, Chicago, IL, USA) for 10 s. The slurry was filtered through a 100 μm steel mesh to produce a smooth milk-like beverage. The oat beverage was stored in glass bottles at 4 °C for no more than 48 h after production.

Oat crisps were prepared using a 1200 W microwave (General Electric, Boston, MA, USA) to dehydrate an oat slurry, which was produced by mixing oats and sucralose-sweetened water in the same procedure as the oat beverage. Differently sweetened oat slurries were microwaved in a 200 mm × 200 mm glass pan for 15 min. The semi-dry oat sheet was then flipped and a 12.7 mm circular cutter was used to cut crisps out of the sheet. The cut crisps were then microwaved for an additional 2 min. The oat crisps were weighed to ensure that each crisp weighed 1.2 ± 0.1 g. The crisps were then cooled in air for 15 min before being vacuum-sealed in plastic and stored at room temperature until served. 

### 2.3. Study Timeline

After an initial consent visit to confirm eligibility for the study, participants visited the sensory lab twice: once after a habitual night and once after a curtailed night of sleep. The lab visits occurred at least one week apart on the same weekday and time (±30 min). Sensory testing transpired during 1 h timeslots between the hours of 7:00–10:00 on weekdays. Participants were assigned a timeslot as close to their habitual wake-time as possible. Participants were not directed to adhere to specific protocols during the time gain by sleep curtailment in order to allow for free-living conditions. The sleep condition sequence was randomly assigned during the consent visit. A sleep curtailment of 33% was determined by centering the self-reported habitual sleep duration and equally reducing bed and wake time in order to minimize circadian rhythm effects while still inducing sleepiness [47]. For example, if the curtailment was 2 h, the participant was required to go to bed 1 h later and wake up 1 h earlier. The study was designed to assess change in hedonic perception under free-living conditions, and therefore, partial sleep curtailment was utilized in place of total sleep deprivation [47]. 

### 2.4. Consent Visit

Participants completed several validated questionnaires during the consent visit. The Pittsburgh Sleep Quality Index (PSQI) [48], Perceived Stress Scale (PSS) [49], and the General Food Craving Questionnaire—Trait version (G-FCQ-T) [50] were used to determine subjective sleep, perceived stress, and general food craving traits, respectively. A relationship between food cravings and reward sensitivity has been reported previously [51] and, thus, food cravings were measured to aid in interpretation of findings. Participants may not have been aware that their sleep habits were abnormal, and thus, the PSQI scores were used to confirm that participants met the criteria for the study. Anthropometrics were also measured for use as covariates. Body mass index (BMI) and percent body fat (% BF) were measured using bioelectrical impedance (TBF-400, Tanita, Arlington Heights, IL, USA).

Objective sleep measures were collected using the Zmachine (General Sleep, Columbus, OH, USA). Participants were trained on how to use the Zmachine at the consent visit. The Zmachine records a single channel (A_1_–A_2_) of electroencephalography (EEG) and uses a scoring algorithm to discriminate between light sleep (LS), slow wave sleep (SWS), REM sleep, and waking states. The Zmachine has been reported to have significant agreement with polysomnography (PSG) [52]. To ensure that participants complied with the assigned protocol, they were instructed to wear the Zmachine 30 min before the predetermined bedtime assigned to them.

To ensure that participants would be fasted after both sleep conditions, they were told to not eat or drink anything other than water between their wake time and their laboratory visit. Additionally, they were told that they would be required to take a “Hydrogen Breath Test”, the results of which would inform the study administrator if they did not follow the fasting instructions. This deceptive procedure was employed to increase compliance with the fasting instructions. The samples were discarded after testing and participants were made aware of the deceit during debriefing.

### 2.5. Laboratory Visits

The testing procedure used was the same for both laboratory visits. EEG data from the previous night’s sleep was promptly uploaded to the Zmachine data viewer upon arrival to the lab. The participant was asked to confirm that the data matched their own recollection of the previous night. If any evidence of machine malfunction, such as significant data loss or disagreement >30 min between participant recollection and machine data readout, then participants returned to the lab no fewer than seven days later with a new sleep recording. Participants then completed the “Hydrogen Breath Test”. 

Before tasting any stimuli, participants completed several validated questionnaires, including the Karolinska Sleepiness Scale (KSS) [53], the Positive Affect-Negative Affect Schedule (PANAS) [54], and the General Food Craving Questionnaire-State version (G-FCQ-S) [50]. These tools were used to measure sleepiness, affect, and food craving state, respectively. Additionally, a 100 mm visual analog scale (VAS) was used to measure hunger with “Extremely Hungry “(0) and “Extremely Full” (100) serving as anchors [55]. The KSS was used along with objective sleep measure to determine the efficacy of the sleep curtailment. The PANAS was used to measure affect changes between the habitual and curtailed sleep conditions to help interpret findings, as changes in affect have been reported to change with sleep curtailment [56] and influence taste perception [57]. Craving states have been found to be associated with sleep duration [4]; therefore, G-FCQ-S data was collected to help aid in interpretation of findings in case cravings were significantly increased by curtailment. Hunger was measured to confirm whether the fasting protocol was effective.

To assess self-perception of the previous night’s sleep quality, participants answered four questions regarding their recollection of the previous night’s sleep [21]. The four questions were: “How much sleep did you obtain last night?”, “How deeply did you sleep last night?”, “How would you rate the quality of your sleep last night?”, and “Compared to an average night of sleep, how comfortable were you when sleeping last night?” The sum of the scores from each of these four questions was used as a measure of overall subjective sleep quality. 

### 2.6. Sensory Evaluation 

RedJade Sensory Software (RedJade, Redwood Shores, CA, USA) was used to manage sensory data collection. All data collection took place at the Michigan State University sensory laboratory. Participants were required to wear nose-clips during sucralose solution tastings but not when consuming oat products. For the sucralose-in-water tasting, participants were instructed to taste the whole cup (10 mL of sample) and expectorate all samples. For the oat product evaluation, the amount served was normalized to 5 kcal, that is, oat crisps were always served in 1.2 ± 0.1 g quantities (5 kcal) and oat beverage was always served in 10 mL quantities (5 kcal). Oat beverage was served cool at 7 °C and while oat crisps and sucralose solutions were served at room temperature (23 °C). Participants did not expectorate oat products. The sensory evaluation consisted of hedonic evaluation of the sucralose solutions and sweet preference testing followed by evaluation of the oat beverage and oat crisps in a random order.

The solutions and products were assessed by presenting a range of five different concentrations of sweetness of each product identified with three-digit blinding codes in a random order. For the sucralose solutions, participants rated their liking of each solution on a 15 cm VAS scale with anchors at 0 (dislike extremely), 7.5 (neutral) and 15 (like extremely). For the oat products, participants rated their overall liking, sweetness liking, flavor liking and texture liking on an identical 15 cm line scale, in that order. In food acceptance tests, it is typical for participants to rate the overall liking of a product, followed by rating a series of product attributes, such as flavor and texture [58]. Additionally, participants were asked to rate how intensely they perceived the sweetness to be on a 15 cm VAS scale with anchors at 0 (not at all intense), 7.5 (no label) and 15 (extremely intense) for both sucralose solutions and oat products. Following the tasting of a sample, there was a 45 s forced wait period in which the participant was required to rinse three times with filtered water. There were three two-minute breaks after every five samples. In total, participants tasted between 10 and 15 sucralose solutions, 5 oat beverages, and 5 oat crisps at each testing visit. 

A modified version of the Monell forced-choice paired comparison protocol [59] was used for preference testing per the methods previously described in Szczygiel et al. 2019 [21]. This version of the protocol reduces the two highest concentrations from the Monell protocol—24% and 36% *w*/*v*—to 18% and 24% *w*/*v*. respectively. The modification to the original protocol was made in order to reduce the possibility of off-tastes when high concentrations of sucralose were used. This modification was used to determine preferred sucralose concentration previously [21]. 

### 2.7. Statistical Analysis

SAS version 9.4 (SAS Institute, Cary, NC, USA.) was used to analyze data. In all analyses, findings were treated as statistically significant if *p* < 0.05 and data are presented as the mean ± standard deviation unless stated otherwise. Overall and attribute liking and intensity scores were plotted against sweetener concentration. The best fit linear functions for each plot were calculated in Excel (Microsoft, Redmond, WA, USA) and the slope of that function became the “Slope” variables used in several analyses. 

A hierarchical cluster analysis (HCA) was conducted in XLstat (Addinsoft, Paris, France) using the five liking scores for each concentration of sucralose in water in order to classify participants into sweet liking phenotypes [35]. HCA is recommended as an objective strategy for classifying study participants into sweet liking phenotypes [36]. Three clusters were identified. Due to the limited sample size, the inverted U-shape responders and sucralose dislikers were grouped into a single “non-liker” group to be used as a fixed factor in further analysis. 

A mixed model was used to determine differences in liking and intensity responses. Sucralose concentration (*n* = 5, 0.004% *w*/*v* −0.094% *w*/*v*), sleep treatment (*n* = 2, curtailed and habitual) food form (*n* = 2, oat beverage and oat crisp), and SLP (*n* = 2, likers and non-likers) were the main fixed factors used throughout the analysis. Participant and interactions between the main fixed factors were included as random factors in all the models. No significant sex, sequence, or period effects were observed in the initial models for sweet taste preference (*p* > 0.05). Therefore, the data for both sexes were pooled and neither sequence nor period were used in any further analysis. Data collected after both nights of sleep, such as PANAS scores or hunger rating, were analyzed using paired *t*-tests and corrected for multiple comparisons using false discovery rate (FDR) with a threshold of *q* = 0.05, which is a strategy used to minimize the risk of type-1 error [2,60]. 

## 3. Results

### 3.1. Participants

Demographics and anthropometrics for the participants are reported in Table 2. Forty-one non-obese participants finished the study. Participants were primarily white (*n* = 27) and female (*n* = 26). Anthropometric measures as well as G-FCQ-T, PSS, and PSQI scores were not correlated with sucralose preference and therefore, were not used in any further analysis (*p* > 0.05). 

### 3.2. Summary of Curtailment

A 34.9% reduction in TIB resulted in restriction of TST, LS, and REM (*p* ≤ 0.001 for all) but not SWS (Table 3). Sleepiness was significantly increased after sleep curtailment, as evidenced by the KSS score increase (*p* < 0.001). Participants reported that the previous night’s sleep was shorter than needed and of a reduced quality (*p* < 0.001). Curtailment reduced perceived sleep quality (*p* < 0.001), but sleep was rated “about average” or higher after both sleep treatments. Participants did not perceive a difference in “deepness” or “comfort” between the two nights.

### 3.3. Summary of Affect, Cravings and Hunger

Curtailment did not result in changes in hunger, negative affect, or food cravings—neither the composite score nor any of the five factors (Table 4). However, curtailment resulted in a decrease in positive affect (*p* < 0.001).

### 3.4. Sweet Liking Phenotypes

Three sweet liking phenotypes (SLP) were identified by hierarchical cluster analysis (HCA) using the hedonic response to five concentrations of the model system (sucralose-sweetened water) after the habitual night. Members of cluster 1 (*n* = 24), the largest cluster, increasingly liked the stimuli as concentration increased until leveling off at 0.032% *w*/*v* (“likers”). Members of cluster 2 (*n* = 10) displayed an inverted U-shape of liking ratings, which began to decrease after 0.032% *w*/*v* (“inverse U-shape”). Members of cluster 3 (*n* = 8), the smallest cluster, liked solutions less as concentration increased (“dislikers”). After curtailment, there were 26 likers, 11 inverse U-shape, and four dislikers. The number of members in each cluster did not significantly differ after sleep curtailment (Kolmogorov–Smirnov, *p* > 0.05); however, this finding obscures the fact that the SLPs were not entirely stable, as nine participants (22%) changed cluster after sleep curtailment. Seven participants moved from either the inverse U-shape or disliker to the liker cluster and two moved from the liker to the disliker cluster. Due to the small number of participants belonging to clusters 2 and 3 based on the model sucralose solutions after the habitual night, these clusters were combined and will henceforth be referred to as “non-likers” (*n* = 17).

### 3.5. Sweetness Perception in the Model System

Sucralose solution data was analyzed separately from the oat products using a mixed model containing sleep condition, SLP, and the interaction term between the two factors.

#### 3.5.1. Model System Sweet Preference

The preferred concentration from the model system was analyzed to confirm the previously reported SLP-independent increase in preferred sucralose concentration after sleep curtailment and to assess whether the SLPs showed differences in preferred concentration. No interaction was observed between the two factors (F(1,39) = 3.08, *p* = 0.087), confirming that SLP and preferred concentration were independent. A significant main effect of the sleep condition on the preferred concentration of sucralose in solution was observed (F(1,39) = 42.24, *p* < 0.001), signifying an increase in preferred concentration after sleep curtailment regardless of SLP, (0.042 ± 0.028% *w*/*v* after the habitual night and 0.063 ± 0.025% *w*/*v* after the curtailed night). Regardless of sleep condition, sweet likers had a higher preferred concentration (M: 0.067% *w*/*v* SD: 0.022) compared to non-likers (M: 0.031% *w*/*v* SD: 0.021) (main effect for SLP on the preferred concentration of sucralose in solution (F(1,39) = 43.53, *p* < 0.001)). 

#### 3.5.2. Model System Sweet Liking Slopes

Model system sweet liking slopes were analyzed to assess whether sleep curtailment resulted in a change in slope of sucralose liking across the sweetener concentrations and whether changes were independent of SLP. For sucralose liking slope, neither the sleep condition by SLP interaction (F(1,39) = 0.0, *p* = 0.953) nor the main effect of sleep condition were significant (F(1,39) = 2.6, *p* = 0.115), indicating that sucralose slope did not significantly increase in steepness after sleep curtailment, regardless of SLP (habitual slope M: 2.4 liking score/0.1% *w/v* sucralose, curtailed slope M: 3.6 liking score/0.1% *w*/*v* sucralose). A main effect for SLP was observed (F(1,39) = 89.84, *p* < 0.001), confirming the difference in slopes between sweet likers (M: 6.9 liking score/0.1% *w*/*v* sucralose) and sweet non-likers (M: −2.5 liking score/0.1% *w*/*v* sucralose). 

#### 3.5.3. Model System Sweet Liking by Concentration

**Figure 1:**
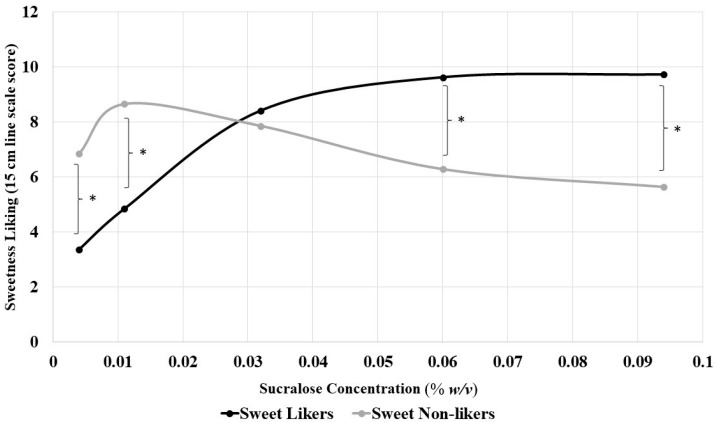
Comparison of sweet liking response, averaged across both sleep conditions, by sweet liking phenotype (sweet likers and non-likers) determined using hierarchical cluster analysis based on liking scores over the range of sucralose solutions after a habitual night of sleep. Likers and non-likers showed distinct patterns of liking with sweet likers showing higher sweetness liking at 0.06% and 0.094% *w/v* sucralose and lower sweetness liking at 0.004% and 0.011% *w*/*v* sucralose, regardless of sleep condition (* *p* < 0.001 for all).

To assess whether liking varied at specific concentrations or overall (across all concentrations) after sleep curtailment, sucralose concentration was added as a five-level fixed factor to the model. No tertiary interactions were observed (*p* > 0.05). Sleep curtailment did not result in significant changes in sweet liking by concentration for sucralose solutions, as evidenced by neither the interaction terms nor the main effects for sleep condition showing significance in the model (*p* > 0.05). Differences in sweetness liking between the SLPs depended on sucralose concentration (sucralose concentration by SLP interaction, F(4,156) = 37.09, *p* < 0.001) (Figure 1). Regardless of the sleep condition, sweet likers reported lower sweet liking ratings for the two lowest concentrations (0.004% *w*/*w*, 0.011% *w*/*v*, *p* < 0.001 for both) and higher sweet liking ratings for the two highest concentrations of model sucralose solutions (0.06% *w*/*v*, 0.094% *w*/*v p* < 0.001 for both), with no difference in liking ratings for the middle concentration (0.032% *w*/*v*), compared to sweet non-likers, confirming significant differences in hedonic responses between likers and non-likers at low and high concentrations.

### 3.6. Hedonic Response in the Oat Product Systems

A four-factor mixed model containing sleep condition, food form, sucralose concentration, and SLP and interactions up to the tertiary level was used to test the primary hypotheses. No tertiary interactions were observed for any oat product models (*p* > 0.05).

#### 3.6.1. Oat Product Sweetness Intensity

**Figure 2:**
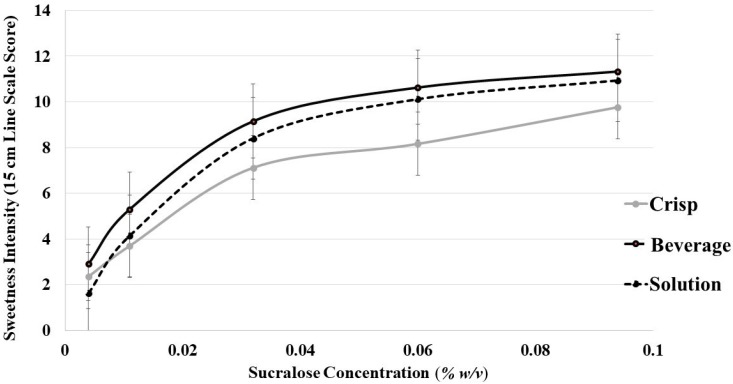
Sweet intensity perception over the range of sucralose concentrations for sucralose solutions, oat beverage, and oat crisps. Sweetness intensity was perceived as higher in oat milk compared to oat crisps, regardless of degree of sweetness (*p* < 0.001). The error bars represent the standard error of the mean.

Sweetness intensity was measured to confirm previous findings that sleep curtailment does not increase sweet taste intensity perception and to assess whether the products were perceived as iso-sweet at each sucralose concentration across the systems used. It was confirmed that sweet intensity perception was not altered after sleep curtailment, as evidenced by neither the interaction terms nor the main effects for sleep condition showing significance in the model (*p* > 0.05). The second concern, whether iso-sweetness between the products was achieved, was assessed by adding sucralose solution intensity scores to the food form factor and testing the sucralose concentration by food form interaction term in the mixed model. This term was not significant (F(4,156) = 1.8, *p* = 0.126), confirming that differences in intensity were similar across the sweetener levels for the food forms and the sucralose (Figure 2). Further, intensity perception did not differ between the SLPs at each sucralose concentration (SLP by sucralose concentration, F(4,12) = 0.69, *p* = 0.614), regardless of sleep condition and food form. However, there was a significant main effect of food form effect on sweetness intensity (F(1,40) = 75.1, *p* < 0.001), signifying that sweetness was more intense for oat beverage compared to oat crisps regardless of sucralose concentration (Figure 2).

#### 3.6.2. Oat Product Liking Slopes

Oat product liking slopes were analyzed to assess whether sleep curtailment resulted in changes in patterns of hedonic response across a range of sweetness levels. Liking slopes for sweetness liking, flavor liking, texture liking, and overall liking were analyzed using a three-factor mixed model containing sleep condition, food form, and SLP and interactions up to the tertiary level. No tertiary interactions were observed (*p* > 0.05). No significant binary interactions were observed between the factors for overall, sweetness, or flavor slopes (*p* > 0.05). The lack of interactions indicates that the three main effects are independent of one another. 

**Figure 3:**
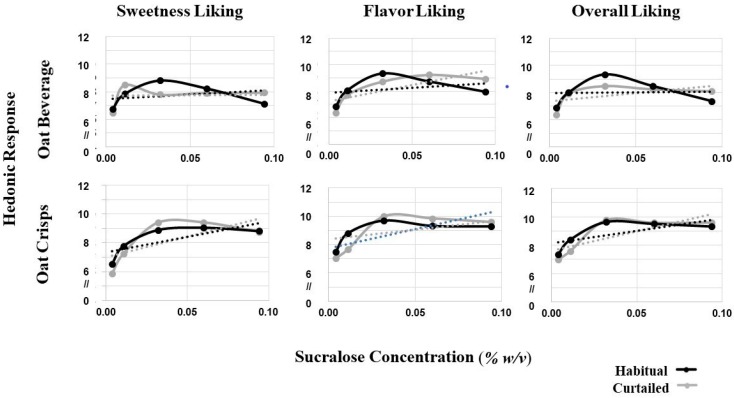
Comparisons between liking responses for different hedonic measurements assessed with a 15 cm visual analog scale for the oat crisp and oat beverage. A significant main effect of sleep was observed for both flavor (*p* = 0.017) and overall liking slopes (*p* = 0.047), indicating overall and flavor liking slopes were significantly steeper after curtailment for both oat products. No effect was observed for sweetness. No interaction between sleep condition and food form was observed. A significant food form effect on the overall (*p* = 0.026) and sweetness liking (*p* = 0.003) slopes was observed, indicating a steeper slope for oat crisps compared to oat beverage regardless of sleep condition.

Several main effects were observed across the hedonic attributes measured. First, a main effect of sleep condition was present for the flavor liking slope (F(1,39) = 11.38, *p* = 0.017) and the overall liking slope (F(1,39) = 4.21, *p* = 0.047), but not for the sweetness or texture liking slope (*p* > 0.05), which demonstrates that overall and flavor liking slopes were steeper after sleep curtailment (Figure 3). The main effect of food form on the slope for overall (F(1,40) = 5.34, *p* = 0.026) and sweetness liking F(1,40) = 9.72, *p* = 0.003) indicate steeper overall and sweetness liking slopes for the oat crisps compared to the oat beverage regardless of sleep condition. A main effect of food form on slope of flavor liking was not observed. The main effect of SLP on liking slopes was significant for slopes of overall (F(1,39) = 9.9, *p* = 0.003), sweetness liking (F(1,39) = 12.7, *p* = 0.001), and flavor (F(1,39) = 7.78, *p* = 0.008), meaning that positive and negative sweet liking slopes for sweet likers and non-likers, respectively extended to both flavor and overall liking for the oat products. 

#### 3.6.3. Oat Product Liking by Concentration

Similarly to the model system “liking by concentration” analysis, sucralose concentration was added as a five-level fixed factor to the model to assess whether liking varied at specific concentrations or overall (across all concentrations). Sleep condition did not interact with concentration (*p* > 0.05), indicating that sleep curtailment did not produce statistically significant differences in the immediate hedonic response at each concentration. Further, the sleep condition by SLP interaction was not significant for any independent variable in this model, providing further evidence that the effect of sleep curtailment on hedonic response did not depend on SLP. 

For sweetness, flavor, and overall liking, no sleep condition by food form interactions was observed (*p* > 0.05). However, a sleep condition by food form interaction was observed for texture (F(4,156) = 7.5, *p* = 0.006), indicating that sleep curtailment resulted in a decrease in texture liking for oat crisps only, regardless of concentration and SLP. Texture liking data for the two oat products were separated and texture liking after a curtailed and habitual night were compared using a reduced mixed model containing only sleep condition and SLP as factors. For the oat beverage, no significant effects of sleep curtailment were observed. For oat crisps, an interaction between sleep condition and SLP was observed (F(1,39) = 21.16, *p* < 0.001), signifying that sweet non-likers were driving differences in texture liking after sleep curtailment. A further analysis revealed a decrease in texture liking in sweet non-likers after sleep curtailment (Habitual: M: 10.5, SD 3.2, Curtailed: M: 9.1, SD: 3.4, *p* = 0.021), but not for sweet likers (Habitual: M: 8.4 SD 2.9, Curtailed: M: 8.8, SD: 3.0, *p* > 0.05).

## 4. Discussion

The primary objective of this study was to determine whether sleep curtailment influences hedonic perception of complex foods, with a focus on sweet taste. The secondary objective was to assess whether these changes are moderated by food form or SLP. Hedonic responses to multiple dimensions of sucralose solutions and sucralose-sweetened liquid and solid oat products were assessed after both a night of habitual sleep and of curtailed sleep. The results from the model solution system are in agreement with our previous findings [21]; preferred sucralose concentration increased and a non-significant increase in liking slope was observed. For the oat products, it was hypothesized that sleep curtailment would result in more pronounced changes in sweet liking and that broader terms such as flavor and overall liking would show significant increases, corresponding with sweetness level. The data partially supports this hypothesis; in oat products, while changes in sweet liking slope were equivalent to model systems, flavor liking and overall liking showed an increase in slope steepness, corresponding with increasing sucralose concentration after sleep curtailment. This finding suggests that participants felt that the products with greater sweetness were holistically preferable to less sweet oat products. Finally, sleep curtailment reduced texture liking of the oat crisps, but not the oat beverage, for sweet non-likers. This suggests a modest effect of sleep on oral somatosensory perception which may only affect sweet non-likers in a solid food model. 

In order to sweeten complex foods across a wide range of sweetness levels, high-intensity non-nutritive sweeteners, such as sucralose [61], can be used to minimize collinear changes in texture [62], aroma [63], and appearance [64] that could occur if iso-sweet quantities of sucrose were used. In a previous study, the effect of sleep curtailment on hedonic response to sucrose and sucralose solutions was determined [21]. Sleep curtailment resulted in an increase in preferred sweetener concentration for both sucrose and sucralose. However, while the effect of sleep curtailment on the slope of sweet liking across a range of sweetness concentrations increased for both sweeteners, the increase was only significant for sucrose. This difference indicates that sucralose perception may be affected by sleep curtailment to a lesser extent than sucrose. This discrepancy may be due to reduced reactivity of brain reward centers in response to non-nutritive sweeteners (NNS) [65]. However, the advantage of controlling the non-taste sensory properties in order to isolate taste changes, which is the main purpose of this study, outweighs potential differences in reward processing between nutritive and NNS. In addition, sucralose is used widely and increasingly in the developed world food supply [66], which means that a large portion of the population is exposed to it on a daily basis. 

The observed increase in flavor and overall liking of the sweeter versions of each food products may play a role in determining food choice and intake after a night of insufficient sleep and suggest that sweeter products were preferable to less sweet products after sleep curtailment. Given that flavor is often the primary determinant of food choice [67] and that the increase in steepness of the flavor liking slope occurred in tandem with a similar shift in overall liking slope, insufficient sleep shows potential to shape both food choice and food intake through changes in hedonic perception. While preferred sweetener concentration was not measured in the oat products, previous work in model systems found that increased slope steepness occurred in tandem with increases in preferred sucralose and sucrose concentration after sleep curtailment [21] and, therefore, it is likely that participants would have selected sweeter versions of the product to consume given the opportunity, although this should be confirmed by future studies. The current study did not test the effects of sucrose in the food systems, but our previous work in model systems suggests that the effects of insufficient sleep are more pronounced for sucrose compared to sucralose, as the sweet liking slope increased significantly after curtailment for sucrose but not sucralose [21]. This discrepancy between the two sweeteners could be due to differential neural processing of nutritive and non-nutritive sweeteners [21,65], which makes hedonic evaluation of sucralose less susceptible to the effect of sleep curtailment. While both sucrose and sucralose activate higher-order brain reward centers in the brain [68], the magnitude of this activation is greater with sucrose exposure [65]. Thus, the results presented likely underestimate the effects of insufficient sleep on sweet taste hedonic responses where nutritively sweetened foods are concerned. In the case of sucrose-sweetened foods, as sleep-curtailed individuals select sweeter foods, these foods tend to be more energy-dense [13] and more likely to promote weight gain. Thus, the observed change in hedonic perception of complex food in this study may contribute to explaining the well-supported relationship between short sleep and obesity [69]. 

Due to the fact that the two oat products were not perceived as iso-sweet, directly comparing the two products to each other, especially in the context of hedonic responses over a range of sweetness levels, is not recommended. The oat beverage was perceived as more sweet compared to the oat crisp regardless of sweetness level, although the differences are much lower than what has been previously reported in similar comparisons between model and complex food systems [25,70], where sweetness intensity perception differed by nearly double. The difference in sweetness intensity perception between the products is likely a result of differences in oral processing of liquid and solid food. Liquids are able to fully and rapidly coat the tongue and, therefore, contact greater numbers of taste receptors, whereas, solids must be masticated and may be swallowed before being fully tasted [70]. Sweetness intensity has also been shown to differ significantly in food products of starkly contrasting temperatures (7 °C and 37 °C) [71]; however, others have concluded that temperature has little effect on perceived sweetness intensity [72], and when it does, the differences are very small. Therefore, the relatively modest difference in the serving temperatures of products in this study (7 °C and 23 °C) make it unlikely that temperature played a significant role in the effects observed.

Sleep curtailment negatively affected texture liking but only for oat crisps and only among non-likers. Sleep curtailment may have decreased texture liking of the oat crisps due to increased oro-facial somatosensory sensitivity after sleep curtailment [33], which may make beverages, semi-solid, or “soft” foods more appealing after sleep curtailment compared to “hard” solid foods. Previous studies reported that sleep restriction increased nociceptor reactivity [73] and oral somatosensory sensitivity [30]. While mechanoreceptors in the mouth are likely the primary contributors to the sense of texture, nociceptors also play an important role, particularly in the instance of “intense pressure,” which may be experienced when consuming foods which shatter or fracture during mastication [74], as with the oat crisps. Beverages and softer foods require less oral processing time and, therefore, decrease satiety compared to foods that necessitate more oral processing, which may lead to excess energy intake and weight gain [75,76]. Therefore, food form could be one factor that mediates the relationship between insufficient sleep and weight gain.

Why the change in texture liking was restricted to sweet non-likers is not known. However, it could be the case that these individuals have increased attention towards the texture of food. Sweet liking patterns might be a single component within a multifaceted collection of attribute liking patterns which determine an individual’s overall liking of a complex food. Overall liking has been described as a function comprised of interactions between hedonic response to individual sensory attributes which are each weighted differently across individuals [77,78]. For example, in one study, while most individuals weighted taste most heavily when considering overall liking, others placed the most importance on texture [77]. It is possible that sweet likers weigh sweetness as a more important factor when considering overall liking and therefore, focus less on other attributes such as texture. This finding suggests that hedonic response to sweet taste may predict hedonic response to other sensory attributes. For example, one study illustrated that a portion of consumers who preferred sweeter chocolate also preferred less cocoa flavor [78]. Furthermore, individual differences in importance placed on specific sensory attributes may moderate the effect of sleep curtailment on food perception, as sleep curtailment affected texture liking for the oat crisps but not the oat beverage. In summary, while SLP does not directly moderate the effect of sleep curtailment on sweet taste, these findings suggest that SLP may be an indicator of other sensory preferences that could be related to changes in food choice after sleep curtailment. 

### Strengths and Limitations

The strengths of this study include the use of novel oat products and sucralose to deliver varying levels of sweetness while minimizing non-sweet sensory differences. The randomized crossover design with a one-week washout period and testing sessions held within 30 min of the previous session on the same day under fasted conditions were also strengths. Additionally, the use of the Zmachine EEG to non-intrusively collect at-home sleep data from participants provided an objective measurement of each sleep condition and confirmation of participant adherence to the prescribed sleep treatment. Limitations of this study include possible fatigue effects from the large sample tasting load per lab visit, the use of sucralose as the sweetener, as opposed to the commonly employed nutritive sweetener, sucrose. Two-minute breaks were instituted between every five samples to minimize fatigue effects. Our findings can only be generalized to foods sweetened with sucralose, which might not represent the primary contributors to weight gain after sleep curtailment. No screening of taste acuity was performed and panelists were not trained to recognize changes in sweetness. Furthermore, the sleep curtailment strategy poses several limitations. Sleep duration in the nights prior to testing was not measured and therefore, the effect of cumulative nights of suboptimal sleep cannot be discounted [79]. Given that participants were not instructed as to how to use time gained by sleep curtailment, differences in the use of light-producing devices during that time may have resulted in differences in circadian rhythm disruption [80]. Finally, the majority of participants in this study were sweet likers, whereas sweet non-likers (comprised of sweet U-shape responders and sweet dislikers) were not well represented. Therefore, it was not possible to compare sweet non-liking phenotypes. A larger sample of sweet U-shape and disliking phenotypes is needed to determine whether these two groups are differentially affected by sleep curtailment. Whether these results apply to individuals with obesity remains to be tested.

## 5. Conclusions

Changes in hedonic responses to both sucralose solutions and sucralose-sweetened oat products were observed after sleep curtailment. In solutions, the sweet liking slope was not significantly increased, but the preferred sucralose concentration was increased after sleep curtailment. In oat products, in agreement with the solution data, the sweetness liking slope did not change, but the slopes of flavor and overall liking functions were steeper after sleep curtailment, corresponding with an increasing sucralose concentration. Given that sucralose concentration and therefore, sweetness, was the only difference between the products, the difference in flavor and overall liking slope suggests that participants felt that the oat products with greater sweetness were holistically preferable. The two SLPs used in this study, likers and non-likers, showed similar changes in hedonic response after sleep curtailment, suggesting that sleep does not differentially affect hedonic responses by phenotype; however, there was one exception. After sleep curtailment, texture liking for sweet non-likers was decreased in oat crisps only, which may point to altered oral somatosensory sensitivity and particular texture salience in sweet non-likers and suggest alternative means by which sleep may influence food choice. These findings represent possible mechanisms by which insufficient sleep leads to weight gain and obesity and signify a possible need to control for the previous night’s sleep quality in affective food sensory studies.

## Figures and Tables

**Table 1:** Macronutrient composition of oat products.

	Oat Beverage	Oat Crisp
Macronutrient	100 kcal	100 kcal
Fat	2 g	2 g
Carbohydrates	18 g	17 g
Protein	3 g	3 g
Crude Fiber	<1 g	<1 g
Moisture	189 g	1 g
Ash	<1 g	<1 g

Stimuli were matched for energy and macronutrient composition. Moisture content differed due to the physical state of the stimuli.

**Table 2:** Anthropometric and demographic summary.

Sex	*n*	%
Male	15	37%
Female	26	63%
Race		
White	27	66%
Asian	13	32%
Other/More than 1	1	2%
Anthropometrics	Mean ± SD	Range
BMI (kg/m^2^)	23.1 ± 3.0	16.4–29.2
BF (%)	24.8 ± 11.8	9.1–35.5
Age (y)	24.1 ± 5.0	18–41
Traits/Habits		
G-FCQ-T (Score)	52.5 ± 18.5	23–117
PSS (Score)	12.1 ± 4.6	3–23
PSQI (Score	3.9 ± 1.1	1–5

Abbreviations: BMI: body mass index, BF: body fat, G-FCQ-T: General Food Craving Questionnaire Trait version, PSS: Perceived Stress Scale, PSQI: Pittsburgh Sleep Quality Index, SD: standard deviation.

**Table 3:** Summary of objective and subjective sleep measures.

		Habitual	Curtailed	% Reduction	*p*-Value	*q*-Value
Objective Sleep Measures (h)	Time in Bed	8.3 ± 0.7	5.4 ± 0.7	34.90%	<0.001	<0.001
	Total Sleep Time	7.2 ± 0.7	4.5 ± 1.0	37.50%	<0.001	<0.001
	Light Sleep	3.8 ± 0.5	2.0 ± 0.8	47.40%	<0.001	<0.001
	REM Sleep	1.9 ± 0.5	1.2 ± 0.4	36.90%	<0.001	<0.001
	Slow Wave Sleep	1.5 ± 0.4	1.4 ± 0.4	6.70%	0.043 ^a^	0.053
Sleepiness (10 pt)	Karolinska Sleepiness Scale	3.5 ± 1.4	5.7 ± 1.6		<0.001	<0.001
Subjective Previous Night’s Sleep Measures (5 pt)	Subjective Sleep Total	13.5 ± 2.0	10.3 ± 2.4		<0.001	<0.001
	How much sleep did you obtain last night?	3.1 ± 0.4	1.5 ± 0.5		<0.001	<0.001
	How deeply did you sleep?	3.6 ± 0.9	3.3 ± 1.0		0.243	0.268
	How would you rate the quality of your sleep	3.8 ± 0.8	2.6 ± 1.0		<0.001	<0.001
	Compared to an average night, how comfortable were you when sleeping last night?	3.0 ± 0.7	2.9 ± 1.0		0.593	0.593

All objective sleep measures were significantly reduced after sleep curtailment. The Karolinska Sleepiness Scale measures sleepiness on a 10-point scale where 1 is “extremely alert” and 10 is “extremely sleepy”. Sleepiness was significantly higher after sleep curtailment. Subjective previous night’s sleep quality was measured using four questions, and the total score was used to represent general subjective sleep quality. Curtailment resulted in a significantly lower total subjective sleep score. *p*-values were obtained from paired *t*-tests, and *q*-values were obtained by correcting *p*-values for false discovery rate. ^a^ After false discovery rate correction, the difference between SWS after a habitual and curtailed night was no longer significant.

**Table 4:** Summary of state-dependent measures.

Measure	Factor	Habitual	Curtailed	*p*-Value	*q*-Value
Hunger	Hunger (100 mm VAS)	67.1 ± 10.24	65.5 ± 10.3	0.916	0.916
G-FCQ-S (0–15 per factor)	Total	44.2 ± 9.7	46.2 ± 12.3	0.429	0.687
	F1-Desire to Eat	6.1 ± 2.0	6.1 ± 2.2	0.948	0.916
	F2-Anticipation to positive reinforcement	8.9 ± 2.0	9.5 ± 2.7	0.232	0.618
	F3-Anticipation to negative reinforcement	11.2 ± 1.8	11.1 ± 2.6	0.859	0.916
	F4-Obsessive preoccupation	6.6 ± 2.4	7.4 ± 3.0	0.124	0.496
	F5-Craving as a physiological state	9.1 ± 2.0	9.4 ± 2.7	0.405	0.687
PANAS	Positive Affect	23.6 ± 2.0	17.6 ± 6.4	<0.001	<0.001
	Negative Affect	12.8 ± 3.9	13.2 ± 4.3	0.539	0.719

Positive affect was significantly decreased after sleep curtailment, whereas, hunger, food craving, and negative affect were not. Larger numbers indicate a greater response. For example, positive affect is higher after a habitual night compared to a curtailed night. FDR correction, shown as *q*-values, did not change the significance of any comparisons. Abbreviations: VAS: Visual Analog Scale, G-FCQ-S: General Food Craving Questionnaire State Version, PANAS: Positive Affect Negative Affect Schedule, F1-5: General Food Craving Questionnaire State Version Factors 1–5.

## References

[1] Szczygiel E.J., Cho S., Tucker R.M. (2018). Characterization of the relationships between sleep duration, quality, architecture and chemosensory function in non-obese females. Chem. Senses.

[2] Szczygiel E.J., Cho S., Snyder M.K., Tucker R.M. (2019). Associations between chemosensory function, sweet taste preference, and the previous night’s sleep in non-obese males. Food Qual. Prefer..

[3] Smith S.L., Ludy M.-J., Tucker R.M. (2016). Changes in taste preference and steps taken after sleep curtailment. Physiol. Behav..

[4] Lv W., Finlayson G., Dando R. (2018). Sleep, food cravings and taste. Appetite.

[5] Simon S.L., Field J., Miller L.E., DiFrancesco M., Beebe D.W. (2015). Sweet/dessert foods are more appealing to adolescents after sleep restriction. PLoS ONE.

[6] Nedeltcheva A.V., Kilkus J.M., Imperial J., Kasza K., Schoeller D.A., Penev P.D. (2009). Sleep curtailment is accompanied by increased intake of calories from snacks. Am. J. Clin. Nutr..

[7] Markwald R.R., Melanson E.L., Smith M.R., Higgins J., Perreault L., Eckel R.H., Wright K.P. (2013). Impact of insufficient sleep on total daily energy expenditure, food intake, and weight gain. Proc. Natl. Acad. Sci. USA.

[8] Hanlon E.C., Andrzejewski M.E., Harder B.K., Kelley A.E., Benca R.M. (2005). The effect of REM sleep deprivation on motivation for food reward. Behav. Brain Res..

[9] Greer S.M., Goldstein A.N., Walker M.P. (2013). The impact of sleep deprivation on food desire in the human brain. Nat. Commun..

[10] Benedict C., Brooks S.J., O’Daly O.G., Almèn M.S., Morell A., Åberg K., Gingnell M., Schultes B., Hallschmid M., Broman J.-E. (2012). Acute sleep deprivation enhances the brain’s response to hedonic food stimuli: An fMRI study. J. Clin. Endocrinol. Metab..

[11] Demos K.E., Sweet L.H., Hart C.N., McCaffery J.M., Williams S.E., Mailloux K.A., Trautvetter J., Owens M.M., Wing R.R. (2017). The effects of experimental manipulation of sleep duration on neural response to food cues. Sleep.

[12] St-Onge M.-P., Wolfe S., Sy M., Shechter A., Hirsch J. (2014). Sleep restriction increases the neuronal response to unhealthy food in normal-weight individuals. Int. J. Obes..

[13] Drewnowski A. (1999). Intense sweeteners and energy density of foods: Implications for weight control. Eur. J. Clin. Nutr..

[14] Drewnowski A., Mennella J.A., Johnson S.L., Bellisle F. (2012). Sweetness and food preference. J. Nutr..

[15] Yamamoto T. (2003). Brain Mechanisms of sweetness and palatability of sugars. Nutr. Rev..

[16] Chen X., Gelaye B., Williams M.A. (2014). Sleep characteristics and health-related quality of life among a national sample of American young adults: Assessment of possible health disparities. Qual. Life Res..

[17] Hales C.M., Carroll M.D., Fryar C.D., Ogden C.L. (2017). Prevalence of Obesity among Adults and Youth: United States, 2015–2016.

[18] Furihata R., Uchiyama M., Suzuki M., Konno C., Konno M., Takahashi S., Kaneita Y., Ohida T., Akahoshi T., Hashimoto S. (2015). Association of short sleep duration and short time in bed with depression: A Japanese general population survey. Sleep Biol. Rhythm..

[19] Lin C.-L., Lin C.-P., Chen S.-W., Wu H.-C., Tsai Y.-H. (2018). The association between sleep duration and overweight or obesity in Taiwanese adults: A cross-sectional study. Obes. Res. Clin. Pract..

[20] Hogenkamp P.S., Nilsson E., Chapman C.D., Cedernaes J., Vogel H., Dickson S.L., Broman J.-E., Schiöth H.B., Benedict C. (2013). Sweet taste perception not altered after acute sleep deprivation in healthy young men. Somnologie.

[21] Szczygiel E.J., Cho S., Tucker R.M. (2019). Multiple dimensions of sweet taste perception altered after sleep curtailment. Nutrients.

[22] Tanaka T., Hong G., Tominami K., Kudo T. (2018). Oral fat sensitivity is associated with social support for stress coping in young adult men. Tohoku J. Exp. Med..

[23] Huber J. (1974). The Psychophysics of Taste: Perceptions of Bitterness and Sweetness in Iced Tea.

[24] Mazur J., Drabek R., Goldman A. (2018). Hedonic contrast effects in multi-product food evaluations differing in complexity. Food Qual. Prefer..

[25] Drewnowski A., Shrager E.E., Lipsky C., Stellar E., Greenwood M.R.C. (1989). Sugar and fat: Sensory and hedonic evaluation of liquid and solid foods. Physiol. Behav..

[26] Tan S.-Y., Tucker R.M. (2019). Sweet taste as a predictor of dietary intake: A systematic review. Nutrients.

[27] Dhillon J., Running C.A., Tucker R.M., Mattes R.D. (2016). Effects of food form on appetite and energy balance. Food Qual. Prefer..

[28] Gujar N., Yoo S.-S., Hu P., Walker M.P. (2011). Sleep deprivation amplifies reactivity of brain reward networks, biasing the appraisal of positive emotional experiences. J. Neurosci..

[29] Krause A.J., Simon E.B., Mander B.A., Greer S.M., Saletin J.M., Goldstein-Piekarski A.N., Walker M.P. (2017). The sleep-deprived human brain. Nat. Rev. Neurosci..

[30] Kamiyama H., Iida T., Nishimori H., Kubo H., Uchiyama M., Laat A.D., Lavigne G., Komiyama O. (2019). Effect of sleep restriction on somatosensory sensitivity in the oro-facial area: An experimental controlled study. J. Oral Rehabil..

[31] Schloegl H., Percik R., Horstmann A., Villringer A., Stumvoll M. (2011). Peptide hormones regulating appetite—Focus on neuroimaging studies in humans. Diabetes/Metab. Res. Rev..

[32] Auvray M., Spence C. (2008). The multisensory perception of flavor. Conscious. Cogn..

[33] Roehrs T., Hyde M., Blaisdell B., Greenwald M., Roth T. (2006). Sleep loss and REM sleep loss are Hyperalgesic. Sleep.

[34] Barr R.G., Pantel M.S., Young S.N., Wright J.H., Hendricks L.A., Gravel R. (1999). The response of crying newborns to sucrose: Is it a “sweetness” effect?. Physiol. Behav..

[35] Iatridi V., Hayes J.E., Yeomans M.R. (2019). Reconsidering the classification of sweet taste liker phenotypes: A methodological review. Food Qual. Prefer..

[36] Iatridi V., Hayes J.E., Yeomans M.R. (2019). Quantifying sweet taste liker phenotypes: Time for some consistency in the classification criteria. Nutrients.

[37] Yeomans M.R., Tepper B.J., Rietzschel J., Prescott J. (2007). Human hedonic responses to sweetness: Role of taste genetics and anatomy. Physiol. Behav..

[38] Asao K., Miller J., Arcori L., Lumeng J.C., Han-Markey T., Herman W.H. (2015). Patterns of sweet taste liking: A pilot study. Nutrients.

[39] Kim J.-Y., Prescott J., Kim K.-O. (2014). Patterns of sweet liking in sucrose solutions and beverages. Food Qual. Prefer..

[40] Mennella J.A., Pepino M.Y., Reed D.R. (2005). Genetic and environmental determinants of bitter perception and sweet preferences. Pediatrics.

[41] Bachmanov A.A., Bosak N.P., Floriano W.B., Inoue M., Li X., Lin C., Murovets V.O., Reed D.R., Zolotarev V.A., Beauchamp G.K. (2011). Genetics of sweet taste preferences. Flavour Fragr. J..

[42] Garneau N.L., Nuessle T.M., Mendelsberg B.J., Shepard S., Tucker R.M. (2018). Sweet liker status in children and adults: Consequences for beverage intake in adults. Food Qual. Prefer..

[43] Holt S.H.A., Cobiac L., Beaumont-Smith N.E., Easton K., Best D.J. (2000). Dietary habits and the perception and liking of sweetness among Australian and Malaysian students: A cross-cultural study. Food Qual. Prefer..

[44] Wiet S.G., Beyts P.K. (1992). Sensory characteristics of sucralose and other high intensity sweeteners. J. Food Sci..

[45] Kim M.-Y., Cho H.-Y., Park J.-Y., Lee S.-M., Suh D.-S., Chung S.-J., Kim H.-S., Kim K.-O. (2005). Relative sweetness of sucralose in beverage systems and sensory properties of low calorie beverages containing sucralose. Korean J. Food Sci. Technol..

[46] Reis F., De Andrade J., Deliza R., Ares G. (2016). Comparison of two methodologies for estimating equivalent sweet concentration of high-intensity sweeteners with untrained assessors: Case study with orange/pomegranate juice. J. Sens. Stud..

[47] Dinges D.F., Pack F., Williams K., Gillen K.A., Powell J.W., Ott G.E., Aptowicz C., Pack A.I. (1997). Cumulative sleepiness, mood disturbance, and psychomotor vigilance performance decrements during a week of sleep restricted to 4–5 hours per night. Sleep.

[48] Buysse D.J., Reynolds C.F., Monk T.H., Berman S.R., Kupfer D.J. (1989). The Pittsburgh sleep quality index: A new instrument for psychiatric practice and research. Psychiatry Res..

[49] Cohen S., Kamarck T., Mermelstein R. (1983). A Global measure of perceived stress. J. Health Soc. Behav..

[50] Cepeda-Benito A., Gleaves D.H., Williams T.L., Erath S.A. (2000). The development and validation of the state and trait food-cravings questionnaires. Behav. Ther..

[51] Meule A., Kübler A. (2014). Double trouble. Trait food craving and impulsivity interactively predict food-cue affected behavioral inhibition. Appetite.

[52] Kaplan R.F., Wang Y., Loparo K.A., Kelly M.R., Bootzin R.R. (2014). Performance evaluation of an automated single-channel sleep–wake detection algorithm. Nat. Sci. Sleep.

[53] Kaida K., Takahashi M., Åkerstedt T., Nakata A., Otsuka Y., Haratani T., Fukasawa K. (2006). Validation of the Karolinska sleepiness scale against performance and EEG variables. Clin. Neurophysiol..

[54] Watson D., Clark L.A., Tellegen A. (1988). Development and validation of brief measures of positive and negative affect: The PANAS scales. J. Personal. Soc. Psychol..

[55] Merrill E.P., Kramer F.M., Cardello A., Schutz H. (2002). A comparison of satiety measures. Appetite.

[56] Franzen P.L., Siegle G.J., Buysse D.J. (2008). Relationships between affect, vigilance, and sleepiness following sleep deprivation. J. Sleep Res..

[57] Noel C., Dando R. (2015). The effect of emotional state on taste perception. Appetite.

[58] Popper R., Rosenstock W., Schraidt M., Kroll B.J. (2004). The effect of attribute questions on overall liking ratings. Food Qual. Prefer..

[59] Mennella J.A., Lukasewycz L.D., Griffith J.W., Beauchamp G.K. (2011). Evaluation of the Monell forced-choice, paired-comparison tracking procedure for determining sweet taste preferences across the lifespan. Chem. Senses.

[60] Glickman M.E., Rao S.R., Schultz M.R. (2014). False discovery rate control is a recommended alternative to Bonferroni-type adjustments in health studies. J. Clin. Epidemiol..

[61] Binns N.M. (2003). Sucralose—All sweetness and light. Nutr. Bull..

[62] Cheer R.L., Lelievre J. (1983). Effects of sucrose on the rheological behavior of wheat starch pastes. J. Appl. Polym. Sci..

[63] Van Boekel M.A.J.S. (2006). Formation of flavour compounds in the Maillard reaction. Biotechnol. Adv..

[64] Ashoor S.H., Zent J.B. (1984). Maillard browning of common amino acids and sugars. J. Food Sci..

[65] Frank G.K.W., Oberndorfer T.A., Simmons A.N., Paulus M.P., Fudge J.L., Yang T.T., Kaye W.H. (2008). Sucrose activates human taste pathways differently from artificial sweetener. NeuroImage.

[66] Sylvetsky A.C., Rother K.I. (2016). Trends in the consumption of low-calorie sweeteners. Physiol. Behav..

[67] MacFie H.J.H., Meiselman H.L. (2012). Food Choice, Acceptance and Consumption.

[68] Green E., Murphy C. (2012). Altered processing of sweet taste in the brain of diet soda drinkers. Physiol. Behav..

[69] Cappuccio F.P., Taggart F.M., Kandala N.-B., Currie A., Peile E., Stranges S., Miller M.A. (2008). Meta-analysis of short sleep duration and obesity in children and adults. Sleep.

[70] Alley R.L., Alley T.R. (1998). The influence of physical state and color on perceived sweetness. J. Psychol..

[71] Calvino A.M. (1986). Perception of sweetness: The effects of concentration and temperature. Physiol. Behav..

[72] Schiffman S.S., Sattely-Miller E.A., Graham B.G., Bennett J.L., Booth B.J., Desai N., Bishay I. (2000). Effect of temperature, pH, and ions on sweet taste. Physiol. Behav..

[73] Kundermann B., Spernal J., Huber M.T., Krieg J.-C., Lautenbacher S. (2004). Sleep deprivation affects thermal pain thresholds but not somatosensory thresholds in healthy volunteers. Psychosom. Med..

[74] Engelen L., Bilt A.V.D. (2008). Oral physiology and texture perception of semisolids. J. Texture Stud..

[75] De Graaf C. (2011). Why liquid energy results in overconsumption. Proc. Nutr. Soc. USA.

[76] James B. (2018). Oral processing and texture perception influences satiation. Physiol. Behav..

[77] Moskowitz H.R., Krieger B. (1995). The contribution of sensory liking to overall liking: An analysis of six food categories. Food Qual. Prefer..

[78] De Kermadec F.H., Durand J.F., Sabatier R. (1997). Comparison between linear and nonlinear PLS methods to explain overall liking from sensory characteristics. Food Qual. Prefer..

[79] Van Dongen H.P.A., Maislin G., Mullington J.M., Dinges D.F. (2003). The cumulative cost of additional wakefulness: Dose-response effects on neurobehavioral functions and sleep physiology from chronic sleep restriction and total sleep deprivation. Sleep.

[80] Wever R.A., Polášek J., Wildgruber C.M. (1983). Bright light affects human circadian rhythms. Pflug. Arch..

